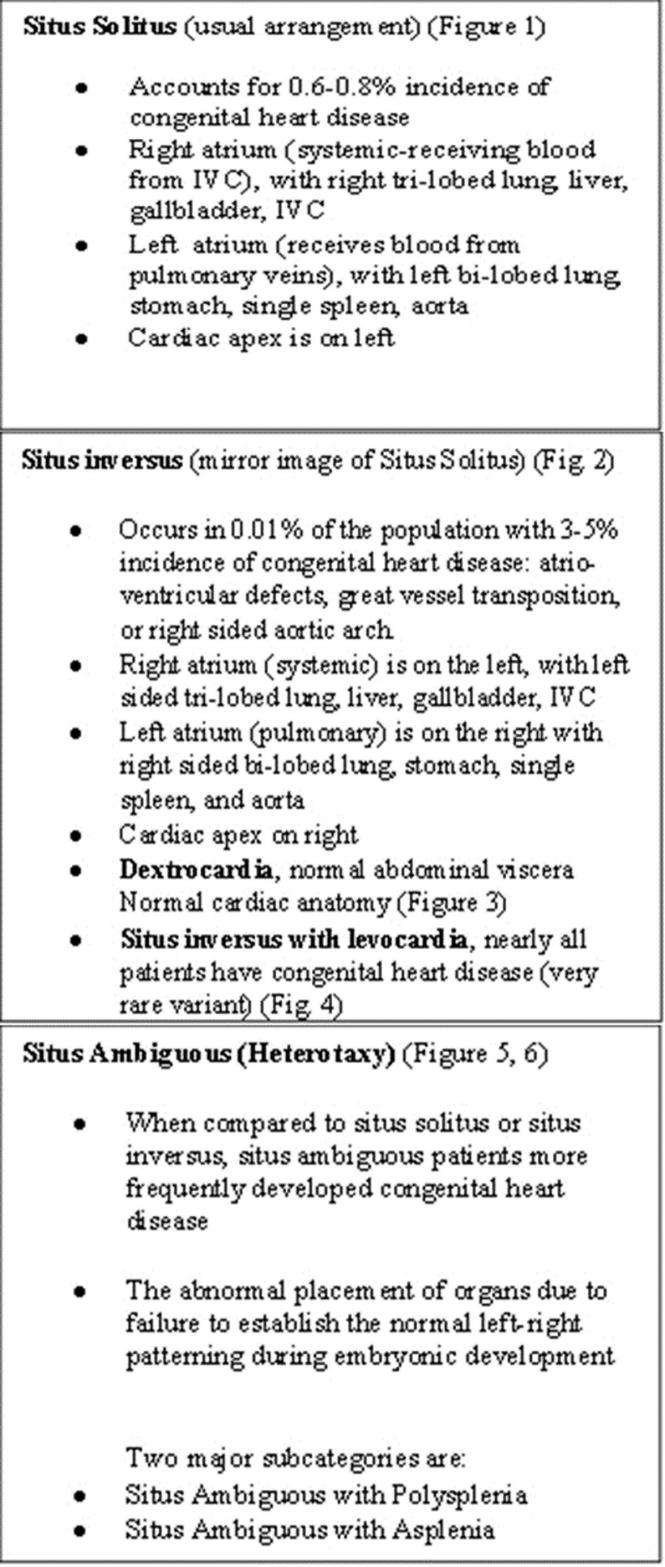# The twisted body: a look into heterotaxy

**DOI:** 10.1186/1532-429X-15-S1-T8

**Published:** 2013-01-30

**Authors:** Ronald B Williams, Moneal Shah, Sahadev T Reddy, June Yamrozik, Mark Doyle, Robert W Biederman

**Affiliations:** 1 Cardiac MRI, Allegheny General Hospital, Pittsburgh, PA, USA

## Background

Throughout the literature, cardiac MRI (CMR) has become an important diagnostic tool in evaluating congenital cardiac abnormalities. In heterotaxy syndrome, the axis of the body during fetal development fails to rotate correctly resulting in complex cardiovascular abnormalities. We hypothesize that CMR aids in the detection of anatomical and physiological anomalies presented in patients with heterotaxy syndrome and its variants. We further hypothesize that CMR permits imaging planes and spatial resolution limited in other imaging modalities.

## Methods

All patients underwent CMR using SSFP, DIR, and/ or PVM via routine VLA, HLA, LVOT, and additional non-orthogonal views when necessary to depict complex anatomy and physiology.

## Results

In a retrospective review of approximately 4500 patient studies from 2007-2011, six (6) patients were found to have cardiac anomalies associated with heterotaxy syndrome: Dextrocardia (1); Situs Inversus Totalis (1); Levocardia with abdominal situs inversus (1), Interrupted IVC without heterotaxy associated asplenia or polyspenia (1), Interrupted IVC with heterotaxy associated asplenia or polysplenia, and right sided aorta (1). In the patient with the right sided aorta, initial diagnosis was double arch versus vascular ring, seen on outside study. CMR demonstrated absent double aortic arch or vascular ring. In the patient with situs totalis, echo imaging failed to provide a diagnosis. CMR revealed no abnormalities other than anatomical malrotation. In the patient with interrupted IVC and polysplenia she was found to have persistent left SVC (PLSVC) draining into the coronary sinus, with hepatic, renal, mesenteric veins draining into the hemi-azygous that drained into this LSVS. The second interrupted IVC, with azygous continuation, showed PLSVC that entered into the dilated sinus, and hepatic veins with direct right atrial connection.

In echocardiography, there are limitations are in the echo acoustic windows, and depth in both TEE or TTE. CT, image acquisition is limited to transverse imaging, relying on post reconstruction. CMR provides orthogonal, oblique imaging, not limited to the traditional anatomical acquisitions in echo or CT.

## Conclusions

CMR provides additive value in patients with heterotaxy syndrome via combination of both anatomic and physiologic imaging providing a compelling rationale for its central role in defining the clinical impact of this syndrome. Knowledge of diverse heterotaxy presentations is important for the CMR technologist, often first to recognize the syndrome, having the initial opportunity to utilize dedicated imaging to define its' manifestations.

## Funding

Internal

**Figure 1:**
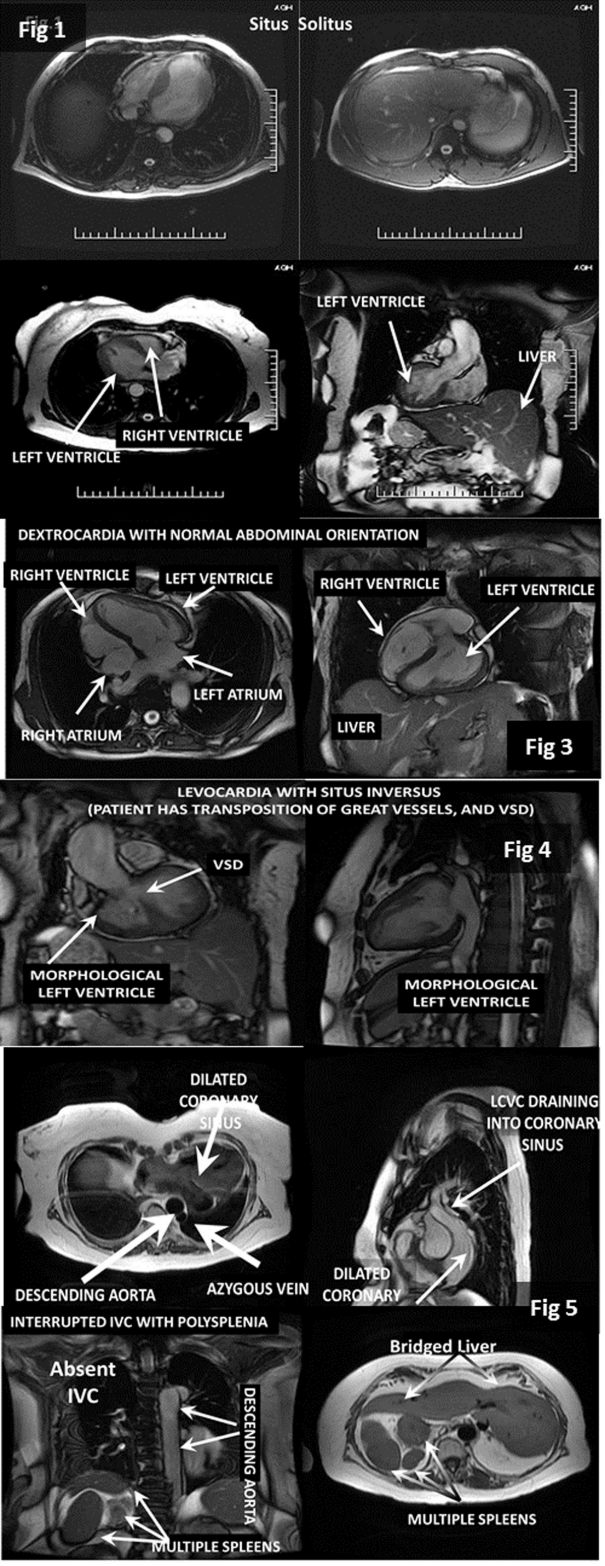


**Figure 2:**